# Herbal Excipients in Novel Drug Delivery Systems

**DOI:** 10.4103/0250-474X.44587

**Published:** 2008

**Authors:** A. Shirwaikar, Annie Shirwaikar, S. Lakshmana Prabu, G. Aravind Kumar

**Affiliations:** Department of Pharmaceutics, Manipal College of Pharmaceutical Sciences, Manipal-576 104, India; 1 Department of Pharmacognosy, Manipal College of Pharmaceutical Sciences, Manipal-576 104, India

**Keywords:** Polysaccharides, volatile oils, controlled delivery

## Abstract

The use of natural excipients to deliver the bioactive agents has been hampered by the synthetic materials. However advantages offered by these natural excipients are their being non-toxic, less expensive and freely available. The performance of the excipients partly determines the quality of the medicines. The traditional concept of the excipients as any component other than the active substance has undergone a substantial evolution from an inert and cheap vehicle to an essential constituent of the formulation. Excipients are any component other than the active substance(s) intentionally added to formulation of a dosage form. This article gives an overview of herbal excipients which are used in conventional dosage forms as well as novel drug delivery systems.

Excipients are primarily used as diluents, binders, disintegrants, adhesives, glidants and sweeteners in conventional dosage forms like tablets and capsules^1^. As the establishment of toxicity and approval from regulatory authorities poses a problem with synthetic excipients, of late more interest is being shown by researchers in herbal excipients. The drawback posed by heavy metal contamination often associated with herbal excipients is superseded by their lack of toxicity, easy availability, and economic considerations in pharmaceutical industry as compared to their synthetic counterparts. Present day consumers look for natural ingredients in food, drugs, and cosmetics as they believe that anything natural will be more safe and devoid of side effects.

The traditional view that excipients are inert and do not exert any therapeutic or biological action or modify the biological action of the drug substance has changed and it is now recognized that excipients can potentially influence the rate and/or extent of absorption of a drug. As herbal excipients are non toxic and compatible, they have a major role to play in pharmaceutical formulation. Hence, this paper is an attempt to review herbal excipients used in NDDS.

## POLYSACCHARIDES IN PHARMACEUTICALS

Natural polysaccharides are extensively used for the development of solid dosage forms. These polymers of monosaccharides (sugars) are inexpensive and available in a variety of structures with a variety of properties. They are highly stable, safe, non-toxic, and hydrophilic and gel forming in nature. Pectins, starch, guar gum, amylase and karaya gum are a few polysaccharides commonly used in dosage forms. Non-starch, linear polysaccharides remain intact in the physiological environment of the stomach and the small intestine, but are degraded by the bacterial inhabitants of the human colon which make them potentially useful in targeted delivery systems to the colon^2^.

### Pectins:

Pectins are non-starch, linear polysaccharides extracted from the plant cell walls. They are predominantly linear polymers of mainly (1–4)-linked D-galacturonic acid residues interrupted by 1,2- linked L-rhamnose residues with a few hundred to about one thousand building blocks per molecule, corresponding to an average molecular weight of about 50 000 to about 1 80 000^2^. Being soluble in water, pectin is not able to shield its drug load effectively during its passage through the stomach and small intestine. It was found that a coat of considerable thickness was required to protect the drug core in simulated *in vivo* conditions^2^. Hence the focus was shifted to the development of less soluble derivatives of pectin which get degraded by the colonic microflora. Calcium salts of pectin reduced their solubility by forming an egg-box configuration. To overcome the drawback of high solubility of pectin, mixed films of pectin with ethyl cellulose were investigated as a coating material for colon-specific drug delivery. These films combined the colon specific degradation properties of pectin with the protective properties of the water insoluble polymer ethyl cellulose^2^.

Polymeric hydrogels are widely used as controlled-release matrix tablets. Sungthongjeen *et al*.^3^ investigated the high-methoxy pectin for its potential value in controlled-release matrix formulations. The effects of compression force, ratio of drug to pectin, and type of pectin on drug release from matrix tablets were also investigated. The results of the *in vitro* release studies showed that the drug release from compressed matrix tablets prepared from pectin can be modified by changing the amount and the type of pectin in the matrix tablets. A very low solubility pectin-derivative (pectinic acid, degree of methoxylation 4%) was found to be well suited as an excipient for pelletisation by extrusion/spheronisation. The capacity as an extrusion aid was found to be high; even formulations containing only 20% pectinic acid resulted in nearly spherical pellets. All pectinic acid pellets were mechanically stable, had an aspect ratio of approximately 1.15–1.20 and released 30–60% of a low solubility model drug within 15 min both in simulated gastric fluid (0.1M HCl) and intestinal fluid (phosphate buffer pH 6.8)^4^.

Micro particulate polymeric delivery systems have been suggested as a possible approach to improve the low bioavailability characteristics shown by standard ophthalmic vehicles (collyria). In this context pectin microspheres of piroxicam were prepared by the spray drying technique. *In vivo* tests in rabbits with dispersions of piroxicam-loaded microspheres also indicated a significant improvement of piroxicam bioavailability in the aqueous humour (2.5–fold) when compared with commercial piroxicam eyedrops^5^.

Musabayane *et al*.^6^ investigated the suitability of amidated pectin as a matrix patch for transdermal chloroquine delivery in an effort to mask the bitter taste when orally administered. The results suggest that the pectin-chloroquine patch matrix preparation has potential applications for the transdermal delivery of chloroquine and perhaps in the management of malaria. Calcium pectinate nanoparticles to deliver insulin were prepared as a potential colonic delivery system by ionotropic gelation^7^. In relation to the food industry, folic acid incorporated microcapsules were prepared using alginate and combinations of alginate and pectin polymers so as to improve stability of folic acid. Folic acid stability was evaluated with reference to encapsulation efficiency, gelling and hardening of capsules, capsular retention during drying and storage. The blended alginate and pectin polymer matrix increased the folic acid encapsulation efficiency and reduced leakage from the capsules as compared to those made with alginate alone, they showed higher folic acid retention after freeze drying and storage^8^.

In relation to cosmetics, using citronellal as a model compound, pectin gel formulations were evaluated for controlled fragrance release by kinetic and static methods. These formulations showed a prolonged duration of fragrance release and limitation of fragrance adsorption to the receptor skin layers. The increase in pectin concentrations suppressed the fragrance release by a diffusion mechanism, thereby proving that pectin/calcium microparticles are promising materials for controlled fragrance release^9^.

### Alginates:

**Fig. 1:**
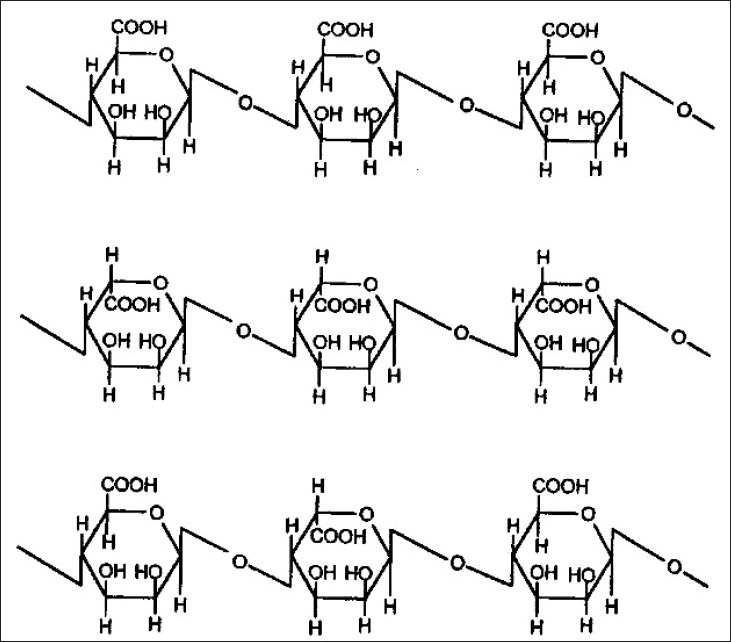
Structure of alginic acid.

Alginates are natural polysaccharide polymers isolated from the brown sea weed (Phaeophyceae). Alginic acid can be converted into its salts, of which sodium alginate is the major form currently used. A linear polymer consisting of D-mannuronic acid and L-guluronic acid residues arranged in blocks in the polymer chain, these homogeneous blocks (composed of either acid residue alone) are separated by blocks made of random or alternating units of mannuronic and guluronic acids. Alginates offer various applications in drug delivery, such as in matrix type alginate gel beads, in liposomes, in modulating gastrointestinal transit time, for local applications and to deliver the bio molecules in tissue engineering applications^10^ (fig. 1).

Bioadhesive sodium alginate microspheres of metoprolol tartrate for intranasal systemic delivery were prepared to avoid the first-pass effect, as an alternative therapy to injection, and to obtain improved therapeutic efficacy in the treatment of hypertension and angina pectoris. The microspheres were prepared using emulsification-cross linking method. *In vivo* studies indicated significantly improved therapeutic efficacy of metoprolol from microspheres, with sustained and controlled inhibition of isoprenaline-induced tachycardia as compared with oral and nasal administration of drug solution^11^.

A new insert, basically consisting of alginates with different hydroxyethylcellulose content was developed to maintain a constant drug level over a certain period in the eye, which cannot be achieved by conventional eye drop application. This study showed good tolerance of the new calcium-alginate-insert applied to the ocular surface for controlled drug release^12^. In order to achieve 24 h release profile of water soluble drugs, sodium alginate formulation matrices containing xanthan gum or zinc acetate or both were investigated. The release of the drug from the sodium alginate formulation containing only xanthan gum was completed within 12 h in the simulated intestinal fluid, while the drug release from the sodium alginate formulation containing only zinc acetate was completed within 2 h in the same medium. Only the sodium alginate formulation, containing both xanthan gum and zinc acetate achieved a 24 h release profile, either in the simulated intestinal fluid or in the pH change medium (pH 1.2). The helical structure and high viscosity of xanthan gum possibly prevent zinc ions from diffusing out of the ranitidine HCL sodium alginate-xanthan gum-zinc acetate matrix so that zinc ions react with sodium alginate to form zinc alginate precipitate with a cross-linking structure. The cross-linking structure might control a highly water-soluble drug release for 24 h^13^.

In a comparative study, alginate formulation appeared to be better than the polylactide-co-glycolide (PLG) formulation in improving the bioavailability of two clinically important antifungal drugs-clotrimazole and econazole. The nanoparticles were prepared by the emulsion-solvent-evaporation technique in case of PLG and by the cation-induced controlled gelification in case of alginate^14^.

### Starches:

**Fig. 2:**
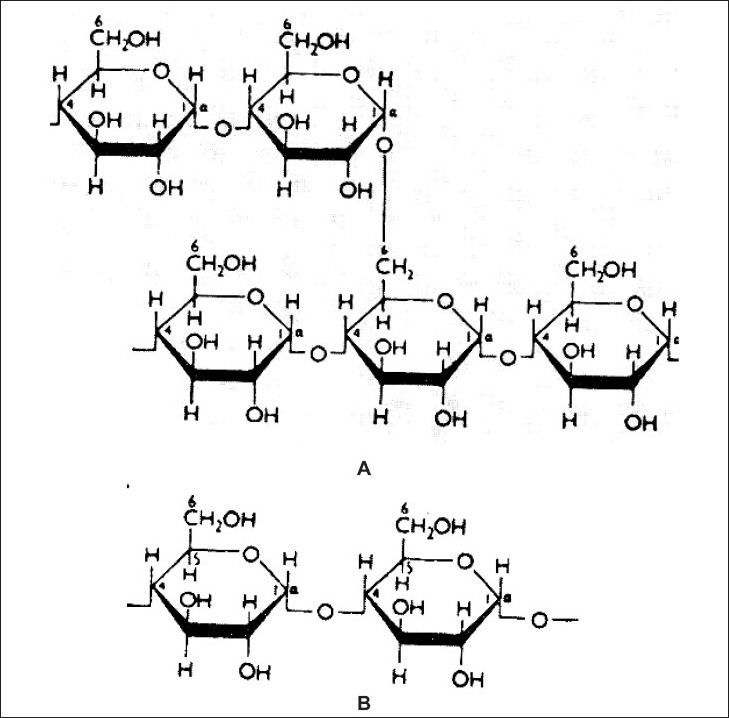
Structures of (A) amylopectin or α- amylase and (B) β-amylose.

It is the principal form of carbohydrate reserve in green plants and especially present in seeds and underground organs. Starch occurs in the form of granules (starch grains), the shape and size of which are characteristic of the species, as is also the ratio of the content of the principal constituents, amylose and amylopectin. A number of starches are recognized for pharmaceutical use (fig. 2). These include maize (*Zea mays*), rice (*Oryza sativa*), wheat (*Triticum aestivum*), and potato (S*olanum tuberosum*)^15^.

Modified starch was tested for general applicability of a new pregelatinized starch product in directly compressible controlled-release matrix systems. It was prepared by enzymatic degradation of potato starch followed by precipitation (retrogradation), filtration and washing with ethanol. The advantages of the material include ease of tablet preparation, the potential of a constant release rate (zero-order) for an extended period of time and its ability to incorporate high percentages of drugs with different physicochemical properties. Release rates from retrograded pregelatinized starch tablets can be enhanced or decreased to the desired profile by different parameters like geometries of the tablet, compaction force and the incorporation of additional excipients^16^.

To deliver proteins or peptidic drugs orally, microcapsules containing a protein and a proteinase inhibitor were prepared. Starch/bovine serum albumin mixed-walled microcapsules were prepared using interfacial cross-linking with terephthaloyl chloride. The microcapsules were loaded with native or amino-protected aprotinin by incorporating protease inhibitors in the aqueous phase during the cross-linking process. The protective effect of microcapsules with aprotinin for bovine serum albumin was revealed *in vitro*^17^.

Acetylating of starch considerably decreases its swelling and enzymatic degradation. Thus, starch-acetate (SA) based delivery systems were tested for controlled drug delivery. It was proved that acetylation of potato starch can substantially retard drug release by preparing and evaluating films of native starch and acetylated starch. Bovine serum albumin (BSA, mol. wt. 68 000), FITC-dextran (mol. wt. 4400), timolol (mol. wt. 332, log P=1.91) and sotalol-HCl (mol. wt. 308, log P=-0.62) were used as model drugs. All the model drugs were released rapidly from the potato starch film in PBS pH 7.4 with and without alpha-amylase in the dissolution medium (t50% varied from 0.17 to 3.37 h). When compared to the potato starch film, all the studied drugs were released at a substantially slower rate from the SA films in the corresponding media^18^. A comparative study was carried out to evaluate drug release from the SA microparticles (SA mps) and SA films.

The average degree of acetyl substitution (DS) per glucose residue in the starch was either 1.9 (SA DS 1.9) or 2.6 (SA DS 2.6). Timolol, calcein and BSA were used as model drugs. This study demonstrated the achievement of slow release of different molecular weight model drugs from the SA mps and films as compared to fast release from the native starch preparations^19^.

## GUMS

Gums are translucent and amorphous substances produced by the plants. Usually pathological products, gums are produced when the plant is growing under unfavorable conditions or when injured. Gums are plant hydrocolloids and may be anionic or non ionic polysaccharides. On hydrolysis gums yield sugar and salts of uronic acid^20^.

### Guar gum:

**Fig. 3:**
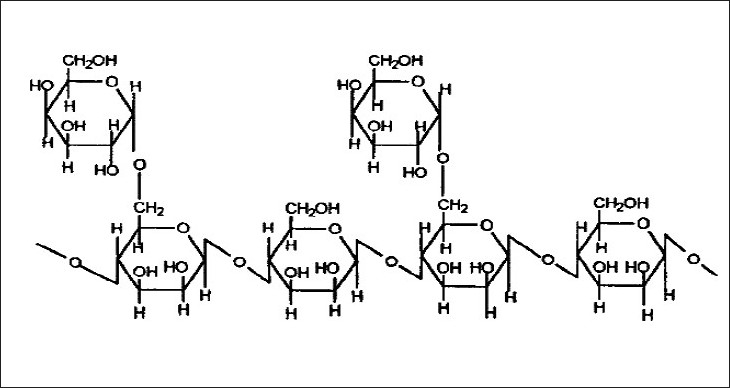
Structure of guar gum.

Guar gum derived from the seeds of *cyamopsis tetragonolobus* (Family Leguminosae) is a naturally occurring galactomannan polysaccharide. It is made up of a linear chain of β-D-mannopyranose joined by β-(1–4) linkage with α-D-galactopyranosyl units attached by 1, 6- links in the ratio of 1:2^2^ (fig. 3).

Guar gum is used in colon-delivery systems due to its drug release retarding property and susceptibility to microbial degradation in the large intestine. Core tablets containing 5-aminosalisylic acid (5–ASA) were prepared by wet granulation with starch paste and were compression coated with coating formulations containing different quantities of guar gum The study confirmed that selective delivery of 5–ASA to the colon can be achieved using guar gum as a carrier in the form of a compression coating over the drug core^21^.

Further, guar gum-based matrix tablets of rofecoxib were prepared for their intended use in the chemoprevention of colorectal cancer. *In vivo* studies showed delayed T_max_, prolonged absorption time and decreased C_max_ indicating that rofecoxib was not released significantly in stomach and small intestine, but was delivered to colon resulting in a slow absorption of the drug and making it available for local action in human colon^22^.

In an attempt to design oral controlled drug delivery systems for highly water-soluble drugs using guar gum as a carrier in the form of three-layer matrix tablets, trimetazidine dihydrochloride was chosen as a model drug because of its high water solubility. Both matrix tablets as well as three layer matrix tablets were prepared and evaluated. The three-layer guar gum matrix tablet provided the required release rate on par with the theoretical release rate for guar gum formulations meant for twice daily administration.

The results indicated that guar gum, in the form of three-layer matrix tablets, is a potential carrier in the design of oral controlled drug delivery systems for highly water-soluble drugs such as trimetazidine dihydrochloride^23^. The same study was carried out by using metoprolol tartrate a model drug with high solubility. The results indicated that guar gum, in the form of three-layer matrix tablets, is a potential carrier in the design of oral controlled drug delivery systems for highly water-soluble drugs such as metoprolol tartrate^24^.

Another water soluble drug, diltiazem HCl has given controlled release comparable with marketed sustained release diltiazem HCl tablets (D-SR tablets), which are prepared in the form of matrix tablets with guar gum using the wet granulation technique^25^.

### Gum acacia:

Gum acacia or gum arabic is the dried gummy exudate obtained from the stem and branches of *Acacia senegal* (Linne) Willdenow and other related species of acacia (Family Leguminosae). The gum has been recognized as an acidic polysaccharide containing D-galactose, L-arabinose, L-rhamnose, and D-glucuronic acid. Acacia is mainly used in oral and topical pharmaceutical formulations as a suspending and emulsifying agent, often in combination with tragacanth. It is also used in the preparation of pastilles and lozenges and as a tablet binder^26^.

Sustained release of ferrous sulfate was achieved for 7 h by preparing gum arabic pellets. Release was further sustained for more than 12 h by coating the pellets with polyvinyl acetate and ethylene vinyl acetate, respectively. An increase in the amount of gum arabic in the pellets decreased the rate of release due to the gelling property of gum arabic. The gel layer acts as a barrier and retards the rate of diffusion of FeSO_4_ through the pellet^27^.

Gum arabic was used as an osmotic, suspending and expanding agent in the preparation of a monolithic osmotic tablet system (MOTS) with two orifices on both side surfaces. Water-insoluble naproxen was selected as the model drug. The optimal MOTS was found to be able to deliver naproxen at a rate of approximately zero order up to 12 h in pH 6.8. Cumulative release at 12 h is 81%, and is independent of environment media and stirring rate. Therefore, these MOTS can be used in the oral drug-controlled delivery field, especially for water-insoluble drugs^28^.

### Karaya gum:

Karaya gum is obtained from *Sterculia urens* (Family sterculiaceae) is a partially acetylated polymer of galactose, rhamnose, and glucuronic acid^26^. Swellable hydrophilic natural gums like xanthan gum and karaya gum were used as release-controlling agents in producing directly compressed matrices. Caffeine and diclofenac sodium, which are having different solubilities in aqueous medium were selected as model drugs. Gum erosion, hydration and drug release studies were carried out using a dissolution apparatus (basket method) at two agitation speeds. In case of xanthan gum neither agitation speed nor drug solubility had any significant effect on water uptake, but matrices with the lower proportion of gum produced a lesser degree of hydration. In contrast, karaya gum displayed a much lower hydration capacity and a higher rate of erosion, both markedly affected by agitation speed. Hence it was concluded that drug release from xanthan and karaya gum matrices depended on agitation speed, solubility and proportion of drug. Both xanthan and karaya gums produced near zero order drug release with the erosion mechanism playing a dominant role, especially in karaya gum matrices^29^. Park *et al*.^30^ showed that mucoadhesive tablets prepared by karaya gum for buccal delivery, had superior adhesive properties as compared to guar gum and was able to provide zero-order drug release, but concentrations greater than 50% w/w may be required to provide suitable sustained release.

### Xanthan gum:

**Fig. 4:**
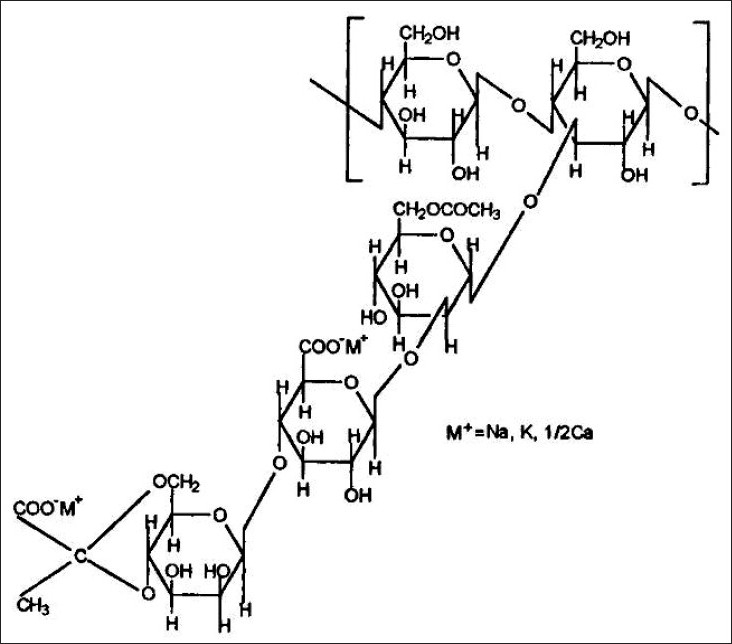
Structure of xanthan gum.

Xanthan gum is a high molecular weight extra cellular polysaccharide produced by the fermentation of the gram-negative bacterium *Xanthomonas campestris*. The primary structure of this naturally produced cellulose derivative contains a cellulosic backbone (β-D-glucose residues) and a trisaccharide side chain of β-D-mannose-β-D-glucuronicacid-α-D-mannose attached with alternate glucose residues of the main chain. The terminal D-mannose residue may carry a pyruvate function, the distribution of which is dependent on the bacterial strain and the fermentation conditions. The non-terminal D-mannose unit in the side chain contains an acetyl function. The anionic character of this polymer is due to the presence of both glucuronicacid and pyruvic acid groups in the side chain^26^ (fig. 4).

In one of the trials, xanthan gum showed a higher ability to retard the drug release than synthetic hydroxypropylmethylcellulose. Xanthan gum and hydroxypropylmethylcellulose were used as hydrophilic matrixing agents for preparing modified release tablets of diltiazem HCl. The amount of hydroxypropylmethylcellulose and xanthan gum exhibited significant effect on drug release from the tablets prepared by direct compression technique. It was concluded that by using a suitable blend of hydroxypropylmethylcellulose and xanthan gum desired modified drug release could be achieved^31^.

Compaction and compression properties of xanthan gum pellets were evaluated and drug release from tablets made of pellets was characterized. Two types of pellets were prepared by extrusion-spheronisation. Formulations included xanthan gum, at 16% (w/w) and diclofenac sodium or ibuprofen, at 10% (w/w) among other excipients. Physical properties of pellets and tablets were analysed. Laser profilometry analysis and scanning electron microscopy of the upper surface and the surface of fracture of tablets revealed that particles remained as coherent individual units after compression process. Pellets showed close compressibility degrees (49.9% for pellets comprising diclofenac sodium and 48.5% for pellets comprising ibuprofen). The release of the model drug from both type of tablets revealed different behaviours. Tablets made of pellets comprising ibuprofen released the model drug in a bimodal fashion and the release behaviour was characterised as Case II transport mechanism (release exponent of 0.93). On the other hand, the release behaviour of diclofenac sodium from tablets made of pellets was anomalous (release exponent of 0.70). For the latter case, drug diffusion and erosion were competing mechanisms of drug release^32^.

By utilizing retention properties of xanthan gum and releasing properties of galactomannan, desire release profile was achieved in delivery of theophylline. Hydrophilic galactomannan is obtained from the seeds of the Brazilian tree *Mimosa scabrella* (Family Leguminosae). The matrices made alone with xanthan gum (X) showed higher drug retention for all concentrations, compared with galactomannan (G) matrices that released the drug too fast. The matrices prepared by combination of both gums were able to produce near zero-order drug release. The XG (conc 8%) tablets provided the required release rate (about 90% at the end of 8 h), with zero-order release kinetics^33^.

### Tragacanth:

This gum is obtained from the branches of *Astragalus gummifer*, Family Leguminosae^20^. Tragacanth when used as the carrier in the formulation of 1- and 3-layer matrices produced satisfactory release prolongation either alone or in combination with other polymers^34^.

## VOLATILE OILS

**Fig. 5:**
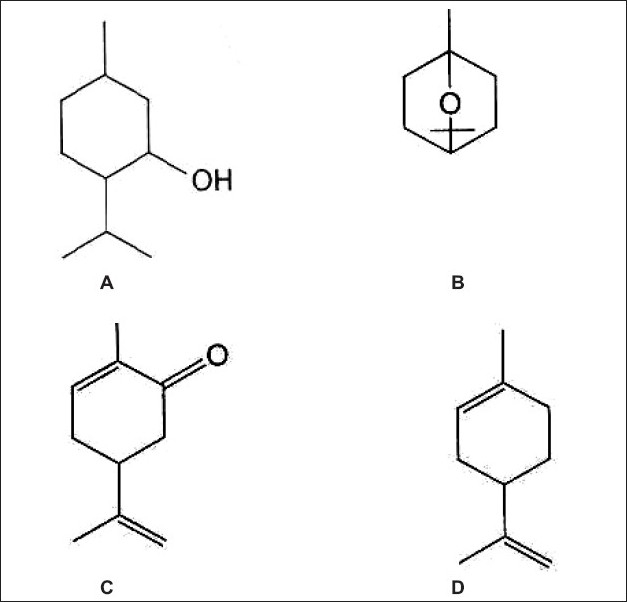
Structures of A. menthol, B. cineole, C. carvone and D.limonene.

Volatile oils are generally mixtures of hydrocarbons and oxygenated compounds derived from these hydrocarbons. Many oils are terpenoid in origin; some of them are aromatic derivatives mixed with terpenes (e.g. cinnamon and clove). A few compounds (e.g. thymol and carvacrol) although aromatic in structure, are terpenoid in origin^15^ (fig. 5).

### Menthol:

Menthol is obtained by steam distillation of the flowering tops of *Mentha piperita* belonging to the family Labiatae. A membrane-moderated transdermal therapeutic system (TTS) of nimodipine using 2%w/w hydroxypropylmethylcellulose (HPMC) gel as a reservoir system containing menthol as penetration enhancer and 60%v/v ethanol-water as solvent system was prepared. The *in vivo* evaluation of nimodipine TTS patch was carried out to find the ability of the fabricated menthol-based TTS patch in providing the predetermined plasma concentration of the drug in human volunteers. The results showed that the menthol-based TTS patch of nimodipine provided steady plasma concentration of the drug with minimal fluctuations with improved bioavailability in comparison with the immediate release tablet dosage form^35^.

Menthol was tested for improving the bioavailability of poorly water-soluble ibuprofen in the rectum with poloxamer. The effects of menthol and poloxamer 188 on the aqueous solubility of ibuprofen were investigated. The poloxamer gel with poloxamer 188 and menthol was found to be a more effective rectal dosage form for ibuprofen^36^. Terpenes such as menthol (fig. 5a), cineole (fig. 5b) and propylene glycol (PG) were tested as chemical enhancers to improve the skin penetration of propranolol. Release and skin permeation kinetics of propranolol from film preparations were examined in *in vitro* studies using a Franz-type diffusion cell. *In vitro* skin permeation studies showed that cineole was the most promising enhancer among the enhancers examined^37^.

### Caraway:

Caraway fruit consists of the dried, ripe fruits of *Carum carvi* (Umbelliferae). The volatile oil consists of the ketone carvone (fig. 5c) and the terpene limonene^15^ (fig. 5d). In another attempt, a limonene-based transdermal therapeutic system (TTS) was prepared to study its ability to provide the desired steady-state plasma concentration of nicorandil in human volunteers. It was concluded that the limonene-based TTS of nicorandil provided the desired plasma concentration of the drug for the predetermined period of time with minimal fluctuations and improved bioavailability^38^.

In a similar manner a carvone based and nerodilol based transdermal therapeutic systems were prepared using nicorandil as a model drug. It was concluded that both TTS of nicorandil provided the desired *in vivo* controlled-release profile of the drug for the predetermined period of time^39^,^40^.

## CONCLUSION

Today the stress is on patient compliance and to achieve this objective there is a spurt in the development of NDDS. As the herbal excipients are promising biodegradable materials, these can be chemically compatible with the excipients in drug delivery systems. In addition herbal excipients are non-toxic, freely available, and less expensive compared to their synthetic counterparts. They have a major role to play in pharmaceutical industry. Therefore, in the years to come, there is going to be continued interest in the natural excipients to have better materials for drug delivery systems.
